# The prevalence of burnout among interns in Riyadh, Saudi Arabia, and its relation to engaging in unethical behaviors

**DOI:** 10.1186/s43045-021-00138-0

**Published:** 2021-10-11

**Authors:** Shatha Ali, Khaldoun Marwa, Malak AlRasheedi, Asma AlSuheel, Mariam Nabila, Madiha Khan

**Affiliations:** 1 King Abdulaziz University Hospital, Jeddah, Saudi Arabia; 2 University of AlMaarefa, Riyadh, Saudi Arabia; 3 Riyadh, Saudi Arabia; 4 Al Yamamah Hospital, Riyadh, Saudi Arabia

**Keywords:** Burnout, Unethical, Exhaustion, Mental health, Medical intern

## Abstract

**Background:**

Burnout is a work-related physical and/or emotional exhaustion among individuals working in the human service sector. This descriptive cross-sectional study aimed to estimate the prevalence of burnout among interns training in different hospitals in Riyadh, Saudi Arabia, and its relation to engaging in unethical behaviors.

**Results:**

The study found a high burnout level in 135 (50%) of the interns with minimal overall engagement in unethical behaviors (5.9%). However, patient-related burnout was the only factor found with a highly significant association to engagement in unethical behaviors (*P*-value < 0.001).

**Conclusions:**

This study demonstrated a high prevalence of burnout among interns with a significant association between patient-related burnout and the engagement in unethical behaviors. That was a worrying sign that needs further evaluation in future research, including the other risk factors, to prevent/improve burnout and to limit the unprofessional behaviors.

## Background

Burnout is a work-related, psychological condition defined as a physical and/or emotional exhaustion and fatigue among individuals working in the human service sector [1]. Burnout can be associated with any job and stress. In medical practice, it refers to the feeling of being overextended, “drained-out,” and “used-up” [2] and is usually a result of prolonged stress or frustration that eventually leads to cynicism, depersonalization, and reduction in personal accomplishments as well as effectiveness [1].

Many studies have discussed burnout prevalence among students and residents, looking for solutions to implicate to minimize the feeling of burnout among them and improve their quality of life, which, in return, would ameliorate their practice and patient care [3, 4]. However, medical interns fall under a different population category, and they have their stressors and concerns that aggravate the feeling of emotional/physical exhaustion and burnout. They are 7th-year medical students holding the Bachelor of Medicine and Bachelor of Surgery degree but lacking the field experience and not yet licensed [5]. They have lots of duties and tasks to do for the first time, and they are expected to cover prolonged working hours, challenging workloads, and on-calls [6].

Not many studies are done to study the stress effect and burnout among interns or house officers, yet it appears that burnout is common among medical interns. Previous studies done in different parts of the world (Brazil, India, the USA, Australia, Mexico, and Pennsylvania) showed a different prevalence of burnout among interns ranging from 18.9 to 57.5% [7–12], with a significantly increased level of burnout among interns at the end of the internship year compared to the start of it (*P* < 0.0001) [9–12].

The reasons behind this high level of burnout and stress among interns are still vague and variable but can be related to the inadequate preparation for practice, financial worries, and sleep deprivation as reported in one study that targeted the interns in Irish hospitals [13] and long working hours and heavy workload as reported in another study among interns in Hong Kong [14]. Moreover, a psychotherapy bulletin discussed the development of interns’ burnout and found that role confusion and unclear power dynamics could be major factors for interns’ burnout [15].

In Saudi Arabia, the internship year is kind of a fateful year for all fresh graduates. Along with the factors previously discussed in the literature, preparation for the Saudi Medical License Exam (SMLE) seems to be a big stressor. SMLE is a cornerstone for the future of every fresh graduate in the Kingdom of Saudi Arabia and needs a lot of effort, time, and dedication. Other factors that can also contribute to the interns’ burnout in Saudi Arabia include a 15-day total annual leave and multiple on-call days every month with no days off during the week in some rotations. These factors participate in the development of emotional/physical exhaustion and burnout of interns, which would eventually affect their quality of life [16].

However, not many studies were done to estimate the prevalence of burnout among medical interns in Saudi Arabia and its related factors. In this study, we aim to estimate the prevalence of burnout among interns training in different hospitals in Riyadh, Saudi Arabia, and its relation to the engagement in unethical behaviors.

## Methods

### Study design

A questionnaire-based descriptive cross-sectional study

### Study area/setting

The study involved almost all governmental and/or private hospitals in Riyadh, Saudi Arabia, that provide internship training programs.

### Study population

#### Sample size

Estimated sample size: the conservative estimate of 50% will be used, *P* = 0.50, a confidence interval of 90%, and a *z* score of 1.64. The margin of error is 0.05

$$n=\frac{p\left(1-p\right){z}^2}{E^2}=\frac{0.50\left(1-0.50\right){1.64}^2}{0.05^2}=\sim 270$$

### Inclusion criteria

- All male and female 7th-year medical students (interns), from all nationalities, training during the period from March 2018 to July 2019, on their expenses or covered by the government or scholarships, in the governmental and/or private hospitals in Riyadh, Saudi Arabia.

### Exclusion criteria

- Interns that were training out of Riyadh city
- 1st- to 6th-year medical students that were rotating in training hospitals

### Sampling technique

Simple random sampling was used in this study; therefore, the data was obtained from interns who were willing and available during the period of data collection.

### Data collection methods

A pre-tested, self-administered questionnaire was distributed randomly via WhatsApp internship, SMLE, and matching groups as an electronic survey among medical interns.

### Data collection tools

The study questionnaire was designed to cover 3 domains: demographic data, burnout, and professional misconduct. Burnout was assessed using the Copenhagen Burnout Inventory, which evaluated three scales: personal burnout scale for general exhaustion, work-related burnout scale for symptoms of work exhaustion, and client-related burnout scale to assess exhaustion related to working with recipients in human services. The burnout scales were administered along with the questionnaire as the second part of it. There was no available pre-tested scale to evaluate the engagement in unethical behaviors, so we gathered the related assessment questions (items) from different previous published articles that discussed unethical behaviors in medical practice. The search provided us with 10 main questions, and we added them under the domain of “professional misconduct” with the same scoring system of the burnout scales. No reliability test was applied to test the assessment of the engagement of unethical behaviors part, but since it was suggested by previously accepted articles, not the authors, we considered them to be objective in the assessment.

### Data analysis

We started data analysis after obtaining 270 filled surveys. All data were statistically analyzed using the Statistical Package for the Social Sciences (SPSS) for Windows, version 21. The results were presented in frequencies and percentages. For burnout scoring, all items had five response categories that were rescaled to a 0–100 metric (the values being 0–25–50–75–100). Scale scores were calculated by taking the mean of the items in that scale. The data about engagement in unethical behaviors were analyzed the same as that of the burnout scale. The odds ratio (OR) and 95% confidence interval (CI) were not calculated, and *P*-value < 0.05 was considered significant.

### Ethical issues

A mandatory consent question was used at the beginning of the survey for their voluntary and anonymous participation in the research, to be filled before proceeding to the survey. If the answer was “do not agree to participate,” the survey ends there with a “thank you” statement. If the answer was “agree to participate,” the person can proceed to the different domains of the survey. The study institutional board review (IRB) is provided by the ethical and research committee of Al Maarefa University. The permission to use the Copenhagen Burnout Inventory was obtained from the developer.

## Results

This study included 270 participants to estimate the prevalence of burnout among interns training in Riyadh hospitals, and its relation to being engaged in unethical behaviors. The majority of respondents were female interns (60%, *N* = 162) and mainly Saudis (87.4%, *N* = 236), and most of them were single (87.4%). One hundred eighteen interns were above 25 years old (43.7%), 103 were 24 years old (38.1%), and only 49 interns were below 24 years old (18.1%). One hundred sixty-eight interns graduated from governmental universities (62.2%). Among the rest, 69 interns’ tuition fees were covered by scholarships, and 33 interns studied on their guardians’ expenses (Table 1).

**Table 1:** Description of the demographic data

		Description (***n*** = 270)
**Age**	22–23, *n* (%)	49 (18.1)
	24, *n* (%)	103 (38.1)
	≥ 25, *n* (%)	118 (43.7)
**Nationality**	Saudi, *n* (%)	236 (87.4)
	Non-Saudi, *n* (%)	34 (12.6)
**Gender**	Male, *n* (%)	108 (40)
	Female, *n* (%)	162 (60)
**Marital status**	Married, *n* (%)	34 (12.6)
	Single, *n* (%)	236 (87.4)
**Is your medical school**	Governmental university	168 (62.2)
	Local private university	93 (34.4)
	Abroad	9 (3.3)
**How did you pay your tuition fee? (*n*** **= 99)**	Scholarship, *n* (%)	69 (67.6)
	Guardian, *n* (%)	33 (32.4)

### Total burnout

Score was found to be high in 50% of the population, mostly in the form of personal (74.1%) and work-related (63.7%) burnout, while the patient-related burnout score was low for 182 interns (67.4%). Engagement in unethical behaviors was found in only 16 interns (5.9%) (Table 2).

**Table 2:** Description of burnout and unethical behaviors’ scores

		Description (*n* = 270)
**Personal burnout**	High, *n* (%)	200 (74.1)
	Low, *n* (%)	70 (25.9)
**Work-related burnout**	High, *n* (%)	172 (63.7)
	Low, *n* (%)	98 (36.3)
**Patient-related burnout**	High, *n* (%)	88 (32.6)
	Low, *n* (%)	182 (67.4)
**Total burnout score**	High, *n* (%)	135 (50)
	Low, *n* (%)	135 (50)
**Unethical behaviors**	High, *n* (%)	16 (5.9)
	Low, *n* (%)	254 (94.1)

### Personal burnout

One hundred four (38.5%) of the participants often felt tired and physically exhausted and 103 (38.1%) reported the feeling of always being emotionally exhausted. Eighty-three (30.7%) of the participants sometimes felt that they could not take it anymore, 91 (33.7%) of the interns sometimes felt worn out, and 94 (34.8%) interns rarely felt weak and susceptible to illness (Table 3).

**Table 3:** Components of burnout and unethical behavior questionnaire

	Never	Rarely	Sometimes	Often	Always
**Personal burnout**	(*n* = 270) *n* (%)				
How often do you feel tired?	2 (0.7)	6 (2.2)	93 (34.4)	104 (38.5)	65 (24.1)
How often are you “physically exhausted”?	4 (1.5)	18 (6.7)	82 (30.4)	104 (38.5)	62 (23)
How often are you “emotionally exhausted”?	3 (1.1)	24 (8.9)	62 (23)	78 (28.9)	103 (38.1)
How often do you think “I cannot take it anymore”?	24 (8.9)	63 (23.3)	83 (30.7)	56 (20.7)	44 (16.3)
How often do you feel worn out?	9 (3.3)	54 (20)	91 (33.7)	74 (27.4)	42 (15.6)
How often do you feel weak and susceptible to illness?	36 (13.3)	94 (34.8)	79 (29.3)	32 (11.9)	29 (10.7)
**Work-related burnout**	(*n* = 270) *n* (%)				
Do you feel worn out at the end of the working day?	8 (3)	29 (10.7)	83 (30.7)	75 (27.8)	75 (27.8)
Are you exhausted in the morning with the thought of “another day at work?”	18 (6.7)	55 (20.4)	83 (30.7)	55 (20.4)	59 (21.9)
Do you feel that every working hour for you is tiring?	23 (8.5)	81 (30)	97 (35.9)	38 (14.1)	31 (11.5)
Do you have enough energy for family and friends during leisure time?	27 (10)	86 (31.9)	88 (32.6)	50 (18.5)	19 (7)
Is your work emotionally exhausting?	15 (5.6)	52 (19.3)	87 (32.2)	62 (23)	54 (20)
Does your work frustrate you?	22 (8.1)	64 (23.7)	100 (37)	47 (17.4)	37 (13.7)
Do you feel burnout because of your work?	18 (6.7)	52 (19.3)	85 (31.5)	68 (25.2)	47 (17.4)
**Patient-related burnout**	(*n* = 270) *n* (%)				
Do you find it hard to work with patients?	44 (16.3)	102 (37.8)	90 (33.3)	22 (8.1)	12 (4.4)
Does it drain your energy to work with patients?	38 (14.1)	87 (32.2)	91 (33.7)	33 (12.2)	21 (7.8)
Do you find it frustrating to work with patients?	50 (18.5)	110 (40.7)	77 (28.5)	19 (7)	14 (5.2)
Do you feel that you give more than you get back when you work with your patients?	44 (16.3)	72 (26.7)	77 (28.5)	40 (14.8)	37 (13.7)
Are you tired of working with patients?	84 (31.1)	89 (33)	60 (22.2)	23 (8.5)	14 (5.2)
Do you sometimes wonder how long you will be able to continue working with patients?	68 (25.2)	69 (25.6)	76 (28.1)	31 (11.5)	26 (9.6)
**Unethical behaviors**	(*n* = 270) *n* (%)				
Do you skip work frequently?	122 (45.2)	92 (34.1)	36 (13.3)	15 (5.6)	5 (1.9)
Have you ever misjudged a situation due to burnout and/or personal involvement?	58 (21.5)	89 (33)	78 (28.9)	29 (10.7)	16 (5.9)
Did you ever try to avoid taking responsibilities due to increased workload?	33 (12.2)	69 (25.6)	98 (36.3)	36 (13.3)	34 (12.6)
Does lack of sleep affect your quality of medical care?	22 (8.1)	42 (15.6)	86 (31.9)	68 (25.2)	52 (19.3)
Did you ever treat any of your patients or their family rudely?	203 (75.2)	34 (12.6)	22 (8.1)	5 (1.9)	6 (2.2)
Did you ever use any inappropriate words while conversing at work with a patient and/or a senior?	222 (82.2)	24 (8.9)	13 (4.8)	8 (3)	3 (1.1)
Did you ever violate hospital rules and regulations?	184 (68.1)	51 (18.9)	21 (7.8)	10 (3.7)	4 (1.5)
Have you ever lied to one of your patients and/or senior?	143 (53)	79 (29.3)	34 (12.6)	6 (2.2)	8 (3)
Have you ever given a patient special care because he/she was of your same tribe, nationality or gender?	217 (80.4)	25 (9.3)	14 (5.2)	6 (2.2)	8 (3)
Have you ever showed less care towards a patient because of his/her low socioeconomic status, untidy appearance or nationality?	232 (85.9)	15 (5.6)	14 (5.2)	5 (1.9)	4 (1.5)

### Work-related burnout

Eighty-three (30.7%) interns sometimes felt worn out at the end of the working day, 97 (35.9%) interns sometimes felt that every working hour is tiring, and 88 (32.6%) interns sometimes had enough energy for family and friends during leisure time. Moreover, 87 (32.2%) interns reported that their work was sometimes emotionally exhausting, 100 (37%) interns mentioned that their work sometimes frustrates them, and 85 (31.5%) interns sometimes felt burnout because of their work (Table 3).

### Patient-related burnout

Ninety (33.3%) interns admitted that they sometimes found it hard to work with patients and 91 (33.7%) of them sometimes felt that working with patients drained their energy. Around 40.7% (110) of the interns rarely found it frustrating to work with patients, 77 (28.5%) interns sometimes felt that they gave more than they got back when they worked with patients, 89 (33%) interns were rarely tired of working with patients, and 76 (28.1%) of them sometimes wondered how long they would be able to continue working with patients (Table 3).

### Engagement in unethical behaviors

The commonest unethical behavior interns were engaged in was letting lack of sleep affect the quality of medical care provided by them (44.5%—120 of the interns always or often did), followed by avoiding to take responsibilities due to increased workload (25.9%—70 of interns always or often did), followed by misjudging a situation due to burnout and/or personal involvement (16.6%—45 of the interns always or often did) (Table 3). The least unethical behaviors interns were engaged in were showing less care towards a patient because of their low socioeconomic status, untidy appearance or nationality (3.4%—9 interns often or always did), using inappropriate words while conversing at work with a patient or a senior (4.1%—11 interns), treating patients/their families rude (4.1%—11 interns), violate hospital rules and regulations (5.2%—14 interns), lying to a senior or patients (5.2%—14 interns), and giving patients special care due to their nationality, gender, or tribe (5.2%—14 interns) (Table 3).

### Factor analysis

Among the factors affecting personal burnout, the male gender was significantly associated with a *P*-value of *P* = 0.011. Moreover, work-related and patient-related burnout were significantly associated with high personal burnout (*P* < 0.001) (Table 4). However, there was no significant association between age, type of medical school interns graduated from, who covered their tuition fees, or interns’ nationalities with the high personal burnout (Table 4).

**Table 4:** Factors related to personal burnout

	Personal burnout (***n*** = 270)		***P*** value*
	High ***n*** (%)	Low ***n*** (%)	
**Age**			
22–23	41 (20.5)	8 (11.4)	0.090
24	73 (36.5)	30 (42.9)	0.346
≥ 25	86 (43)	32 (45.7)	0.694
**Nationality**			
Saudi	173 (86.5)	63 (90)	0.447
Non-Saudi	27 (13.5)	7 (10)	
**Gender**			
Male	71 (35.5)	37 (52.9)	**0.011**
Female	129 (64.5)	33 (47.1)	
**Marital status**			
Married	25 (12.5)	9 (12.9)	0.938
Single	175 (87.5)	61 (87.1)	
**Is your medical school**			
Governmental university	124 (62)	44 (62.9)	0.899
Local private university	72 (36)	21 (30)	0.363
Abroad	4 (2)	5 (7.1)	0.053
**How did you pay your tuition fee? (*n*** **= 99)**			
Scholarship	50 (65.8)	19 (73.1)	0.493
Guardian	26 (34.2)	7 (26.9)	
**Work-related burnout**			
High	157 (78.5)	15 (21.4)	**<0.001**
Low	43 (21.5)	55 (78.6)	
**Patient-related burnout**			
High	81 (40.5)	7 (10)	**<0.001**
Low	119 (59.5)	63 (90)	

*P value = 0.05 is considered significant

Among the factors discussed in this study, only patient-related burnout was significantly associated with high work-related burnout (*P* = 0.002 and *P* < 0.001, respectively) (Table 5).

**Table 5:** Factors related to work-related burnout

	Work-related burnout (***n*** = 270)		***P*** value*
	High ***n*** (%)	Low ***n*** (%)	
**Age**			
22–23	31 (18)	18 (18.4)	0.944
24	63 (36.6)	40 (40.8)	0.496
≥ 25	78 (45.3)	40 (40.8)	0.470
**Nationality**			
Saudi	148 (86)	88 (89.8)	0.372
Non-Saudi	24 (14)	10 (10.2)	
**Gender**			
Male	62 (36)	46 (46.9)	0.079
Female	110 (64)	52 (53.1)	
**Marital status**			
Married	25 (14.5)	9 (9.2)	0.203
Single	147 (85.5)	89 (90.8)	
**Is your medical school**			
Governmental university	104 (60.5)	64 (65.3)	0.430
Local private university	62 (36)	31 (31.6)	0.463
Abroad	6 (3.5)	3 (3.1)	0.851
**How did you pay your tuition fee? (*n*** **= 99)**			
Scholarship	43 (63.2)	26 (76.5)	0.178
Guardian	25 (36.8)	8 (23.5)	
**Patient-related burnout**			
High	78 (45.3)	10 (10.2)	**<0.001**
Low	94 (54.7)	88 (89.8)	

*P value = 0.05 is considered significant

Although being over 25 years old (45.5%), being Saudi (87.5%), being a female (58%), and graduating from a governmental university (64.8%) were all related to having high patient-related burnout, but no factor with significant association to high patient-related burnout was identified (Table 6).

**Table 6:** Factors related to patient-related burnout

	Patient-related burnout (***n*** = 270)		***P*** value*
	High ***n*** (%)	Low ***n*** (%)	
**Age**			
22–23	20 (22.7)	29 (15.9)	0.175
24	28 (31.8)	75 (41.2)	0.137
≥ 25	40 (45.5)	78 (42.9)	0.687
**Nationality**			
Saudi	77 (87.5)	159 (87.4)	0.975
Non-Saudi	11 (12.5)	23 (12.6)	
**Gender**			
Male	37 (42)	71 (39)	0.633
Female	51 (58)	111 (61)	
**Marital status**			
Married	11 (12.5)	23 (12.6)	0.975
Single	77 (87.5)	159 (87.4)	
**Is your medical school**			
Governmental university	57 (64.8)	111 (61)	0.548
Local private university	30 (34.1)	63 (34.6)	0.932
Abroad	1 (1.1)	8 (4.4)	0.279
**How did you pay your tuition fee? (*n*** **= 99)**			
Scholarship	19 (61.3)	50 (70.4)	0.365
Guardian	12 (38.7)	21 (29.6)	

*P value = 0.05 is considered significant

Moreover, only graduation from a university abroad was moderately statistically of significant correlation with total burnout score (*P* = 0.036 and *P* = 0.006, respectively) (Table 7).

**Table 7:** Factors related to total burnout score

	Total burnout score (***n*** = 270)		***P*** value*
	High ***n*** (%)	Low ***n*** (%)	
**Age**			
22–23	26 (19.3)	23 (17)	0.636
24	48 (35.6)	55 (40.7)	0.380
≥ 25	61 (45.2)	57 (42.2)	0.624
**Nationality**			
Saudi	120 (88.9)	116 (85.9)	0.463
Non-Saudi	15 (11.1)	19 (14.1)	
**Gender**			
Male	54 (40)	54 (40)	1.000
Female	81 (60)	81 (60)	
**Marital status**			
Married	17 (12.6)	17 (12.6)	1.000
Single	118 (87.4)	118 (87.4)	
**Is your medical school**			
Governmental university	85 (63)	83 (61.5)	0.802
Local private university	49 (36.3)	44 (32.6)	0.522
Abroad	1 (0.7)	8 (5.9)	**0.036**
**How did you pay your tuition fee? (*n*** **= 99)**			
Scholarship	33 (66)	36 (69.2)	0.727
Guardian	17 (34)	16 (30.8)	

*P value = 0.05 is considered significant

Among the factors assessed in this study, only high patient-related burnout was statistically significantly associated with engagement in unethical behaviors (*P* < 0.001). The demographics and other burnout domains were of no significant statistical value in this regard (Table 8).

**Table 8:** Factors related to unethical behaviors

	Unethical behaviors (***n*** = 270)		***P*** value*
	High ***n*** (%)	Low ***n*** (%)	
**Age**			
22–23	5 (31.3)	44 (17.3)	0.180
24	6 (37.5)	97 (38.2)	0.956
≥ 25	5 (31.3)	113 (44.5)	0.300
**Nationality**			
Saudi	15 (93.8)	221 (87)	0.702
Non-Saudi	1 (6.3)	33 (13)	
**Gender**			
Male	9 (56.3)	99 (39)	0.171
Female	7 (43.8)	155 (61)	
**Marital status**			
Married	3 (18.8)	31 (12.2)	0.435
Single	13 (81.3)	223 (87.8)	
**Is your medical school**			
Governmental university	13 (81.3)	155 (61)	0.106
Local private university	3 (18.8)	90 (35.4)	0.173
Abroad	0 (0)	9 (3.5)	0.444
**How did you pay your tuition fee? (*n*** **= 99)**			
Scholarship	3 (100)	66 (66.7)	0.549
Guardian	0 (0)	33 (33.3)	
**Personal burnout**			
High	13 (81.3)	187 (73.6)	0.769
Low	3 (18.8)	67 (26.4)	
**Work-related burnout**			
High	11 (68.8)	161 (63.4)	0.665
Low	5 (31.3)	93 (36.6)	
**Patient-related burnout**			
High	12 (75)	76 (29.9)	**<0.001**
Low	4 (25)	178 (70.1)	
**Total burnout score**			
High	10 (62.5)	125 (49.2)	0.303
Low	6 (37.5)	129 (50.8)	

*P value = 0.05 is considered significant

## Discussion

To our knowledge, this is the first study estimating the prevalence of burnout among interns in Riyadh, Saudi Arabia, and correlating it to the engagement in unethical behaviors. Globally, burnout during the internship year has been a real concern, and many studies were carried out to estimate its prevalence, dig into the major problems leading to it, and try to find solutions. In this study, we found that 50% of the participants had a high total burnout score, which is almost similar to the result of another study that was carried out in Brazil showing a total burnout prevalence of 57.5% among the interns [7], and another study that estimated the prevalence of total burnout among Australian interns to be 55.9% [10]. These results were a bit higher than those of an institutional study that was carried out in India to estimate the burnout prevalence among Indian interns, and it showed that only 22% of the participants exhibited burnout during their internship training [8]. This could rely on the use of different burnout defining scales, since the Indian study used the Maslach Burnout Inventory scales (emotional exhaustion, depersonalization, personal achievements) while we used the Copenhagen Burnout Inventory scales (personal burnout, work-related burnout, patient/client-related burnout); moreover, the Indian study used all the three sub-scales to define total burnout which resulted in low overall prevalence but the individual scales scored higher rates of burnout, i.e., 34% of subjects exhibited high emotional exhaustion, 46% had high depersonalization, and 77% had low personal accomplishment which is consistent with the rates of this study and previous studies in the literature.

Additionally, burnout was higher among male responders and was significantly correlated with personal burnout (*P* = 0.011); this higher prevalence is inconsistent with the literature findings of female predominance [11]; however, the study that was carried out among Indian interns showed no gender significant relation to burnout [8], which makes it a matter for further investigation in future research.

Besides gender, there were other factors associated with high burnout, including other forms of burnout and graduating from an abroad medical school. It was an interesting finding that graduating from an international medical school lead to more burnout, and it raised the question of whether it was caused by the change of the healthcare system or maladjustment to new culture and expectations.

Moreover, personal burnout (74.1%) and work-related burnout (63.7%) were the main forms of burnout reported among participants. This reaffirmed the findings of a previous longitudinal study that showed a significant increase of personal and work-related burnout in mid internship year, however, subsequently diminished (*P* = 0.0001, *P* = 0.0015, respectively) [10]. It could be explained by the uncertainty of future career, lack of experience, and coping mechanisms especially by the beginning of the internship year.

Engagement in unethical behaviors was found to be generally minimal (5.9%); however, interns with patient-related burnout were more likely to engage in unethical behaviors (*P* < 0.001). So, it was worrying how the compassion fatigue (unmanaged feelings of frustration and growing tired of dealing with patients) may lead the intern to judgmental behavior and lack of empathy, sense of responsibility, and professionalism.

Among the most commonly reported unethical behaviors, poorer quality of care due to lack of sleep (44.5%), trying to avoid work and responsibilities due to high workload (25.9%), and misjudging a situation due to personal involvement/burnout (16.6%) were the commonest unethical behaviors among interns. Accordingly, it was very significant to focus on improving working conditions, distributing duties equally, and offering counseling services to healthcare providers generally.

Despite the high levels of burnout and the correlation of personal burnout with engaging in unethical behaviors, the overall percentage of ethical behaviors were insignificant (*P* = 0.786). The minimal engagement denotes the presence of protective factors, possibly good social support, religious background, or the personal moral system.

There were several limitations in this study, including the capacity of each hospital involved as workload and subsequent burnout might be reflected by the number of interns and the distribution of duties among them. The relationship between unethical behaviors and demographic data was not investigated. Also, the correlation between burnout and other factors, like working conditions, preparation for the Saudi Medical License Exam (SMLE), and social and medical backgrounds, were not reviewed.

## Conclusions

The study demonstrated a high prevalence of burnout among interns training in Riyadh, Saudi Arabia. The factors behind this high prevalence were not explored in our study; however, it is crucial to investigate more about them and grasp the best ways to counteract their effect on the interns in the future. On the other hand, the significant association between patient-related burnout and the engagement in unethical behaviors found in the study was a worrying sign that needs further evaluation to be done, including other risk factors, to find strategies that would limit the unprofessional behavior.

## Data Availability

The datasets used and/or analyzed during the current study are available from the corresponding author on reasonable request.

## Abbreviations

SPSS: Statistical Product and Service Solutions
MBBS: Bachelor of Medicine and Bachelor of Surgery
IRB: Institutional board review
